# Syphilis

**Published:** 1895-12

**Authors:** 


*Syphilis.* By ALFRED COOPER. Second Edition. Edited by EDWARD COTTERELL. Pp. xx., 480. London: J. & A. CHURCHILL. 1895.

We gladly welcome a second edition of the valuable work issued eleven years ago by Mr. Alfred Cooper, who has had exceptional opportunities of gaining a practical experience of the subject on which he writes. The value of the work is further enhanced by being edited by Mr. Cotterell, who from his association with the out-patient department of the London Lock Hospital, is able to deal with the subject as an authority.

Since the publication of the first edition, it has been found necessary to completely revise all the chapters and rewrite several of them. That on "Syphilis and Insanity," in which acknowledgment is made of considerable indebtedness to the writings of Dr. G. H. Savage, is entirely new and is a very valuable one.

The history and geographical distribution of syphilis are amply considered and will be read with much interest. We were particularly pleased with the illustrations, which form a new feature of this edition. Many of them are admirable examples of colour printing, and altogether the typographical dress in which the book is presented is a most attractive one, doing the publishers the greatest possible credit.

We have no hesitation in saying that this work should prove generally interesting, and of very great value to those who have to give practical attention to the subject.

